# Clinical Characteristics and Survival Analysis in Frequent Alcohol Consumers With COVID-19

**DOI:** 10.3389/fnut.2021.689296

**Published:** 2021-06-02

**Authors:** Ricardo Wesley Alberca, Paula Ordonhez Rigato, Yasmim Álefe Leuzzi Ramos, Franciane Mouradian Emidio Teixeira, Anna Cláudia Calvielli Branco, Iara Grigoletto Fernandes, Anna Julia Pietrobon, Alberto Jose da Silva Duarte, Valeria Aoki, Raquel Leão Orfali, Maria Notomi Sato

**Affiliations:** ^1^Laboratorio de Dermatologia e Imunodeficiencias (LIM-56), Departamento de Dermatologia, Faculdade de Medicina FMUSP, Instituto de Medicina Tropica, Universidade de São Paulo, São Paulo, Brazil; ^2^Technical Division of Medical Biology, Immunology Center, Adolfo Lutz Institute, São Paulo, Brazil; ^3^Department of Immunology, Institute of Biomedical Sciences, University of São Paulo, São Paulo, Brazil

**Keywords:** COVID-19, SARS-CoV-2, alcohol, consumption, inflammation

## Abstract

Severe acute respiratory syndrome coronavirus-2 (SARS-CoV-2) infection can generate a systemic disease named coronavirus disease–2019 (COVID-19). Currently, the COVID-19 pandemic has killed millions worldwide, presenting huge health and economic challenges worldwide. Several risk factors, such as age, co-infections, metabolic syndrome, and smoking have been associated with poor disease progression and outcomes. Alcohol drinking is a common social practice among adults, but frequent and/or excessive consumption can mitigate the anti-viral and anti-bacterial immune responses. Therefore, we investigated if patients with self-reported daily alcohol consumption (DAC) presented alteration in the immune response to SARS-CoV-2. We investigated 122 patients with COVID-19 (101 male and 46 females), in which 23 were patients with DAC (18 men and 5 women) and 99 were non-DAC patients (58 men and 41 women), without other infections, neoplasia, or immunodeficiencies. Although with no difference in age, patients with DAC presented an increase in severity-associated COVID-19 markers such as C-reactive protein (CRP), neutrophil count, and neutrophil-to-lymphocyte ratio. In addition, patients with DAC presented a reduction in the lymphocytes and monocytes counts. Importantly, the DAC group presented an increase in death rate in comparison with the non-DAC group. Our results demonstrated that, in our cohort, DAC enhanced COVID-19-associated inflammation, and increased the number of deaths due to COVID-19.

## Introduction

Severe acute respiratory syndrome coronavirus-2 (SARS-CoV-2) infection can generate a severe systemic and multi-organ disease named coronavirus disease-2019 (COVID-19). SARS-CoV-2 has infected and killed millions worldwide (1). Several risk factors, such as co-infections (2), old age, chronic respiratory diseases, and comorbidities (3) have been associated with poor outcomes of COVID-19.

During the pandemic, reports have highlighted the increase in alcohol consumption and the increased risk to trigger or exacerbate depressive and anxious episodes (4). Excessive consumption of alcohol can impair the immune response (5) present a deleterious effect in chronic viral infection (6, 7) and increase the severity and recovery time for respiratory infections (8–11).

The use of substances such as tobacco smoking is an established risk factor for severe COVID-19 (12), but few reports have identified people with alcohol use disorder or daily alcohol consumption (DAC) in their cohorts, with reports identifying no influence on neither drinking alcohol on severity nor death rates in patients with COVID-19 (13, 14). Therefore, we aimed to perform an investigation in our cohort to assess if DAC could influence the disease course and outcome of COVID-19.

## Methods

Patients were recruited fromat a special ward in the university hospital (Hospital das Clinicas da Universidade de São Paulo - HCFMUSP) for moderate and severe patients with COVID-19. Inclusion criteria consisted of a positive diagnosise for COVID-19 by the detection of SARS-CoV-2 RNA in nasopharyngeal swab by reverse-transcriptase PCR (RT-PCR). DAC was determined by self-report, some patients were clinically diagnosed with alcohol-dependency prior to COVID-19, and some reported necessity for DAC of over two drinks.

Patients with and without DAC with type 2 Diabetes Mellitus (DM) and/or systemic arterial hypertension (SAH) were also included. The exclusion criteria were the presence of co-infections, renal or heart diseases, immunodeficiencies, or neoplasia. This investigation was approved by the Ethics Committee of HCFMUSP with approval for the usage of digital data from patients (no. 30800520.7.0000.0068-2020) and was performed in accordance with the 2013 revision of the Declaration of Helsinki. On the first day after hospitalization, EDTA plasma samples were obtained from a single venipuncture, and laboratory analysis was performed at the Central Laboratory of HCFMUSP (Divisão de Laboratório Central—HCFMUSP). Statistical analysis for age and laboratory data was performed using the Mann-Whitney test for comparisons between groups. Survival curve statistical analysis was performed using the Log-rank (Mantel-Cox) test and the Gehan-Breslow-Wilcoxon test with GraphPad Prism 8 software (GraphPad Software, Inc., San Diego, CA).

Were included in this investigation 23 individuals with DAC (18 men and 5 women) and 99 patients with non-DAC (58 men and 41 women) that were hospitalized between June 2020 and 1 December 2020.

## Results

From the 23 DAC patients with COVID-19, 11 had moderate level COVID-19 and were admitted into the general ward (GW) (±48%) and 12 were admitted into the intensive care unit (ICU) (±52%) and needed assisted mechanical ventilation. It is important to highlight that among the 23 DAC patients with COVID-19, one patient has DM (±4%), six patients have SAH (±26%), and five patients have both DM and SAH (±21%).

From the total 99 non-DAC patients with COVID-19, 52 were admitted into the GW (±52%) and 47 were admitted into the ICU (±47%). Among the 99 patients with COVID-19: 4 patients have DM (±4%), 23 patients have SAH (±23%), and 24 patients have both DM and SAH (±24%).

The Patients with non-DAC and DAC did not present any age difference or significant statistical differences in the levels of alanine aminotransferase, aspartate aminotransferase, alkaline phosphatase, total bilirubin, direct bilirubin, indirect bilirubin, glutamyl transferase gamma, creatinine, lactate dehydrogenase, total protein and fractions, albumin, globulin, urea, and D-dimer, prothrombin time (Table 1). Nevertheless, both non-DAC and DAC presented alterations in alanine aminotransferase, aspartate aminotransferase, direct bilirubin, glutamyl transferase gamma, creatinine, lactate dehydrogenase, C-reactive protein (CRP), urea, D-dimer, and the prothrombin time were outside the considered normal range (Table 1).

**Table 1:** Patients' clinical characteristics and laboratory data.

	**Non-DAC (*****N*** **=** **99)**		**DAC (*****N*** **=** **23)**			
	**Mean**	**SEM**	**Mean**	**SEM**	**Reference numbers**	***p*-value**
Male/Female	58/41	18/5				
Age (years)	55.08	1.335	56.38	3.135	–	0.2963
Laboratory data						
Alanine aminotransferase (U/L)	**48.43**	**5.797**	**48.13**	**7.436**	**<41**	**0.9238**
Aspartate aminotransferase (U/L)	**43.44**	**4.149**	**51.42**	**9.261**	**<37**	**0.7681**
Alkaline phosphatase (U/L)	115	18.89	107.2	15.78	40–129	0.7865
Total bllirubin (mg/dL)	0.4538	0.06193	0.4533	0.0485	0.2–1.0	0.3681
Direct bilirubin (mg/dL)	**0.3578**	**0.0604**	**0.3183**	**0.03358**	**<0.3**	**0.6342**
Indirect bilirubin (mg/dL)	0.09385	0.01291	0.03358	0.02393	0.1–0.6	0.2229
Glutamyl transferase gamma (U/L)	**164.9**	**30.59**	**156.6**	**19.33**	**8–61**	**0.4998**
Creatinine (mg/dL)	**2.12**	**0.2077**	**2.042**	**0.3794**	**0.7–1.2**	**0.9659**
Lactate dehydrogenase (U/L)	**457.9**	**33.48**	**451.2**	**55.76**	**135–225**	**0.4321**
**C-reactive protein (mg/L)**	77.54^**^	**9.205**	154.2^**^	**27.97**	**<5.0**	**0.0089**
Total protein and fractions (g/dL)	5.794	0.2328	6.37	0.2574	6.6–8.7	0.1698
Albumin (g/dL)	2.919	0.1046	3.255	0.1895	3.4–4.8	0.2643
Globulin (g/dL)	2.875	0.2065	3.085	0.1464	1.7–3.5	0.3153
Urea (mg/dL)	**75.13**	**5.447**	**85.17**	**12.7**	**10–50**	**0.5556**
D-dimer (ng/mL)	**1963**	**252.2**	**1497**	**175.5**	**<500**	**0.4949**
**Platelets (x 10**^**3**^**/mm**^**3**^**)**	277.7^*^	15.51	206.6^*^	21.89	150–400	0.0474
Prothrombin time (seconds)	**13.99**	**0.3263**	**13.08**	**0.3433**	**9.4–12.5**	**0.1133**
**Activated partial thromboplastin time (seconds)**	38.87^*^	2.408	28.84^*^	1.034	25.1–36.5	0.0169

*P values indicate differences between groups*.

* *p < 0.05 and*

** *p < 0.01 were considered statistically significance. Bold represents values outside reference range. Reference values from Divisão de Laboratorio Central do HC/FMUSP*.

We observed an increase in CRP and a reduction in platelets and activated partial thromboplastin time in patients with DAC in comparison to those with non-DAC (Table 1). This indicates an overall increase in COVID-19 biomarkers, such as urea, D-dimer, and creatinine levels in both groups, but a further increase in CRP levels showed a decrease in platelet counts, which was previously associated with COVID-19 severity (15, 16). The CRP level, especially at early hospitalization, has been associated with the extension of COVID-19-induced lung injury and disease progression, and death (16, 17). Models for predicting the disease outcome of COVID-19 are based on inflammatory markers on the first day of hospitalization (18).

**Figure 1:**
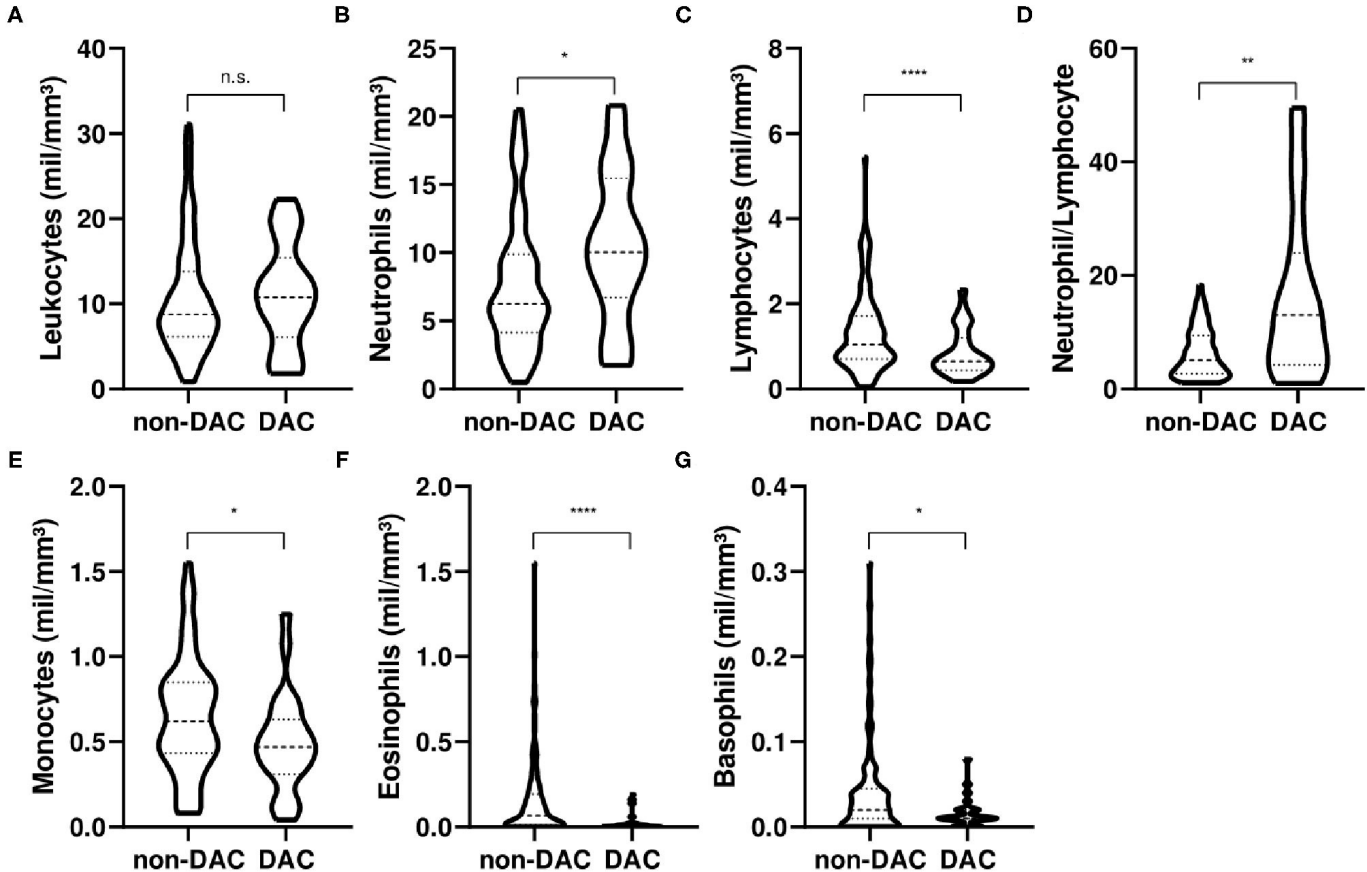
Clinical features of patients with COVID-19. **(A)** Leukocyte counts, **(B)** Neutrophil counts, **(C)** Lymphocyte counts, **(D)** Neutrophil-to-lymphocyte ratio, **(E)** Monocyte counts, **(F)** Eosinophil counts, and **(G)** Basophil counts from the first day of hospitalization due to the SARS-CoV-2 infection. DAC, patients with self-reported daily alcohol consumption (DAC) and COVID-19; Non-DAC, patients without DAC and COVID-19. *p < 0.05, **p < 0.01, ****p < 0.0001, n.s., non-statistically significant difference. Mann-Whitney test used for comparisons. Data collected between June 1, 2020 and December 1, 2020.

Interestingly, although the total leukocyte count was similar among groups (Figure 1A), DAC groups presented an increase in the neutrophil and neutrophil to lymphocyte ratio (Figures 1B,D). Neutrophil count and the neutrophil-to lymphocyte-ratio are also established hallmarks of COVID-19 and are associated with disease severity (19, 20). An increase in neutrophils, as well as neutrophil migration to the lungs, is associated with lung inflammation and immunothrombosis in patients with COVID-19 (21). The neutrophil- to lymphocyte-ratio is a widely used maker for COVID-19 prognoses (19, 20, 22, 23), but the increase in the DAC group was due to both an increase in the neutrophil count (Figure 1B) and a reduction in the lymphocytes count in relation to the non-DAC group (Figure 1C).

Lymphopenia, the reduction in the number of lymphocytes, is common in patients with COVID-19, with a significant reduction in T helper cells, T cytotoxic cells, Natural Killer, and B cells, impacting the anti-SARS-CoV-2 and other co-infections (2, 24, 25). In fact, lymphopenia is also associated with the severity and recovery from COVID-19 (26).

In our cohort, the number of monocytes in patients with DAC was reduced in comparison with patients with non-DAC (Figure 1E). Currently, reports have identified phenotypical alteration on monocytes of patients with COVID-19 (27), with a reduction in the antigen-presenting potential and an increase in the pro-inflammatory markers (28). Nevertheless, the reduction in monocytes of patients with DAC may also indicate a deficient immune response to SARS-CoV-2, as monocytes have a fundamental role in the immune response to pathogenic microorganisms (29).

We identified a reduction in both eosinophils and basophils in the DAC group in comparison with the non-DAC (Figures 1F,G). Low eosinophil and basophil counts have been associated with the worst anti-SARS-CoV-2 response (30).

**Figure 2:**
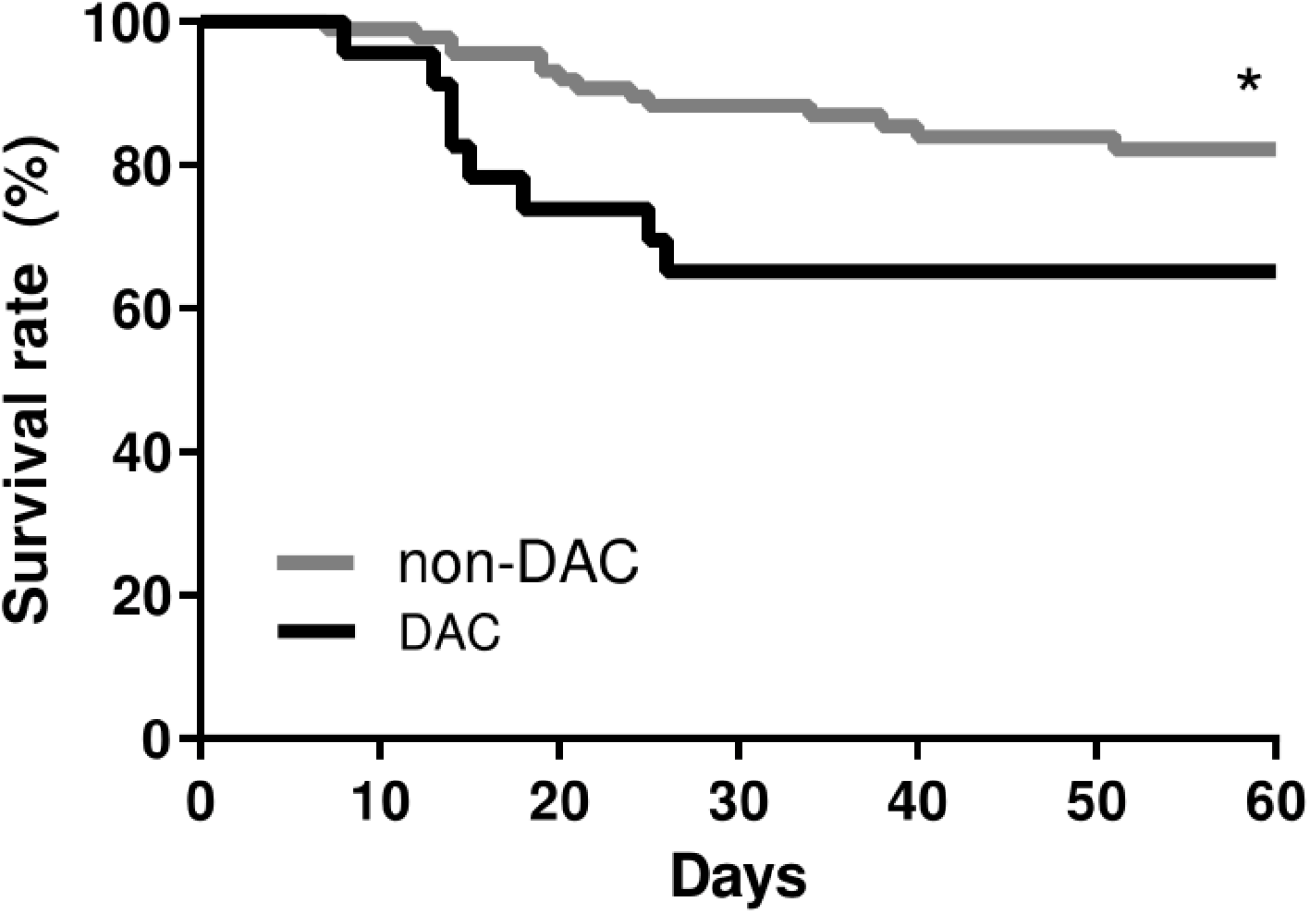
Survival curve of patients with COVID-19. DAC, patients with self-reported DAC and COVID-19; Non-DAC, patients without DAC and COVID-19. *p < 0.05 difference. Comparison using the Log-rank (Mantel-Cox) test and the Gehan-Breslow-Wilcoxon test. Data collected between June 1, 2020 and December 1, 2020.

We failed to identify differences in hospitalization time of the DAC group, which was 29.6 ± 6.1 days and the non-DAC group, which was 29.8 ± 3 days. It is noteworthy that a significant increase in death rate in the DAC group in comparison with the non-DAC group (Figure 2) [*p* = 0.0417 using the Log-rank (Mantel-Cox) test and *p* = 0.0209 using the Gehan-Breslow-Wilcoxon test]. During hospitalization, 8 patients from the DAC group and 14 patients from the non-DAC group passed away due to COVID-19 besides the efforts of the medical staff.

## Discussion

Excessive alcohol consumption can increase the severity and recovery time for infections (8). Several reports have highlighted the negative impact of excessive alcohol consumption on the bacterial respiratory infection (31) and viral respiratory infections such as respiratory syncytial virus (9) and influenza (10).

The increased risk for respiratory viral infections is due to the immune modulation caused by alcohol consumption (8, 10). A previous report identified an increase in neutrophils and tumor necrosis factor-α (TNF) and monocyte chemoattractant protein-1 in the lungs of mice after alcohol consumption (32). Importantly, neutrophil count and TNF levels are associated with severe COVID-19 infection (33). In our cohort, patients with DAC presented a significant increase in the neutrophil and neutrophil to lymphocyte ratio, indicating a possibility that the frequent or excessive alcohol could further increase the severity of the infection caused by SARS-CoV-2.

Another hallmark alteration in chronic alcohol consumption is the reduction in the number and activity of NK cells, T cells, and B cells, decreasing the overall immune response and antibody production (34). This study verified that patients with DAC suffered from a significant reduction in the lymphocyte count in comparison to non-DAC and, that alcohol consumption was further detrimental to the lymphopenia suffered by patients with COVID-19 (22).

Short-term alcohol consumption can also impair the monocyte immune response, with an increase in the production of inflammatory cytokines but a decrease in the activation of the inflammatory complex by microbial components (35). Monocytes isolated from individuals after alcohol consumption present an increase in the activation of the inflammatory complex (inflammasome) and production of pro-inflammatory cytokine, IL-1β (36).

Importantly, patients with COVID-19 also present phenotypical alteration on monocytes, increased pro-inflammatory response, and reduced antigen presentation potential (27, 28). Although the role of monocytes' on COVID-19 is still under investigation, alcohol consumption may exacerbate the dysregulated profile of monocyte on COVID-19.

The long-term consumption of alcoholic beverages has been associated with longer hospitalization and intensive care usage upon respiratory infections (8). In our cohort, we did not identify any differences in hospitalization time between DAC and non-DAC groups.

Alcohol consumption is also associated with a reduction of anti-inflammatory molecules, such as IL-10 (37), which may further aggravate the COVID-19-associated lung inflammation and established dysregulation in the anti-inflammatory immune response (24). Besides, alcohol consumption modulates the gut microbiome (38), and curbs the viral immune response (39), which may expose the individual to SARS-CoV-2 gastrointestinal infection (40, 41), leading to, increased gastrointestinal dysbiosis (42) and more severe respiratory complication due to COVID-19 (43).

Excessive alcohol consumption also impacts the absorption of nutrients, with a reduction in essential minerals and vitamins (44). Chronic alcohol consumption can impair vitamin A (45) and vitamin D (46) levels. Vitamin A deficiency reduces B-and T-cell immune response to influenza in mice (47, 48), and could further enhance the COVID-19-mediated immune dysregulation (24). Vitamin D deficiency has been associated with an increase in respiratory distress syndrome (49), COVID-19 severity (50), and deaths (51).

Although to our knowledge, no investigation on the angiotensin-converting enzyme (ACE2) expression in the lungs has been performed following alcohol consumption, investigations have identified that chronic alcohol consumption can increase the expression of ACE in the blood (52) and angiotensin II type 2 (AT2) receptor in the lungs (53). This leads to the hypothesis that alcohol consumption might modulate the ACE2 receptor, the entry receptor of SARS-CoV-2, in a similar way to other diseases, either increasing or reducing COVID-19 risk (54–56).

It is noteworthy that we identified an increase in mortality in the DAC group in comparison to the non-DAC group due to COVID-19 respiratory complications, which could indicate that frequent alcohol consumption may increase the death risk by COVID-19.

This study possesses two limitations: (1) the quantity of alcohol ingested by members of the DAC group may vary and could be an important variant; (2) it was impossible to isolate DAC from other comorbidities (SAH and/or DM) due to the number of individuals with DAC. Therefore, it is possible that the synergic effect of alcohol consumption with comorbidities influenced the COVID-19 outcome.

## Conclusion

In summary, in our cohort, frequent alcohol consumption alters the COVID-19 clinical characteristics, further increasing pro-inflammatory markers and increasing the mortality risk due to COVID-19-associated complications. Due to the limited number of patients, further investigations should explore the possible synergic effect of alcohol consumption with other comorbidities on COVID-19 severity and mortality.

## Data Availability Statement

The original contributions presented in the study are included in the article/supplementary material, further inquiries can be directed to the corresponding author/s.

## Ethics Statement

The studies involving human participants were reviewed and approved by Ethics Committee of Hospital das Clínicas da Faculdade de Medicina da Universidade de São Paulo—HCFMUSP with approval for the usage of digital data from patients (no. 30800520.7.0000.0068-2020). The patients/participants provided their written informed consent to participate in this study.

## Author Contributions

All authors contributed to data collection, analysis, write and review of the article, and approved the submitted version.

## Conflict of Interest

The authors declare that the research was conducted in the absence of any commercial or financial relationships that could be construed as a potential conflict of interest.
